# Post-marketing safety profile of ganirelix in women: a 20-year pharmacovigilance analysis of global adverse drug event databases (2004–2024)

**DOI:** 10.1186/s40360-025-00920-4

**Published:** 2025-04-22

**Authors:** Yang Yang, Zhiwei Cui, Xiaoshan Feng, Fan Zou, Xiaoling Wu

**Affiliations:** 1 Department of Obstetrics and Gynecology, The Second Affiliated Hospital of Xi’an Jiaotong University, Xi’an Shaanxi, 710004 People’s Republic of China; 2 Department of Obstetrics and Gynecology, The First Affiliated Hospital of Xi’an Jiaotong University, Xi’an, Shaanxi China; 3 Department of Respiratory and Critical Care Medicine, Affiliated Hospital of Zunyi Medical University, Zunyi, China

**Keywords:** Ganirelix, Adverse drug events, Pharmacovigilance, Real-world evidence, FAERS, JADER, Assisted reproductive technology

## Abstract

**Background:**

Ganirelix, a third-generation GnRH antagonist, is widely used in assisted reproductive technology (ART) for rapid pituitary suppression to prevent premature luteinizing hormone (LH) surges. Despite its extensive clinical use, real-world evidence on its safety in large populations remains scarce. This study aimed to evaluate the safety profile of ganirelix by comprehensively analyzing adverse drug events (ADEs) using real-world data from the U.S. Food and Drug Administration Adverse Event Reporting System (FAERS) and the Japan Adverse Drug Event Reporting (JADER) database.

**Methods:**

We extracted ADE data from FAERS (Q1 2004–Q2 2024) and JADER (Q1 2009–Q1 2024). Disproportionality analyses, including reporting odds ratios (ROR), proportional reporting ratios (PRR), Bayesian Confidence Propagation Neural Networks (BCPNN), and Multi-item Gamma Poisson Shrinkage (MGPS), were employed to identify significant associations between ganirelix and ADEs.

**Results:**

In the FAERS database, we identified 1,096 ganirelix-related ADE reports, spanning 26 system organ classes (SOCs). A total of 65 positive signals were detected, including ADEs consistent with drug label such as ovarian hyperstimulation syndrome (OHSS) (*n* = 290, ROR 2462.76, PRR 2168.48, EBGM05 1655.59, IC025 9.18), injection site pain (*n* = 54, ROR 3.99, PRR 3.93, EBGM05 3.13, IC025 0.31), and fetal death (*n* = 6, ROR 21.05, PRR 21.00, EBGM05 10.72, IC025 2.72). Additionally, unexpected signals not listed in the drug label were identified, including ectopic pregnancy (*n* = 7, ROR 33.02, PRR 32.93, EBGM05 17.64, IC025 3.37), maternal exposure before pregnancy (*n* = 30, ROR 76.09, PRR 75.16, EBGM05 74.72, IC025 6.22), dermatitis allergic (*n* = 4, ROR 7.98, PRR 7.97, EBGM05 3.50, IC025 1.33), and bladder tamponade (*n* = 4, ROR 771.47, PRR 770.3, EBGM05 311.57, IC025 7.80). The median time to ADE onset was 13 days. External validation using the JADER database (62 ganirelix-related ADE reports) confirmed four signals, including abortion (*n* = 19), OHSS (*n* = 17), missed abortion (*n* = 9), and fetal death (*n* = 8), aligning with FAERS findings.

**Conclusion:**

This study provides a robust real-world safety evaluation of ganirelix, with findings corroborated by two independent pharmacovigilance databases. While consistent with clinical observations, the identification of unexpected signals warrants further pharmacoepidemiological investigations to confirm these results.

**Supplementary Information:**

The online version contains supplementary material available at 10.1186/s40360-025-00920-4.

## Introduction

Before the introduction of gonadotropin-releasing hormone agonists (GnRHa), approximately 20% of stimulated cycles in in vitro fertilization (IVF) programs were canceled due to premature luteinizing hormone (LH) surges, which compromise follicle quality and maturity, and may result in premature follicular rupture [1]. The use of GnRHa to prevent LH surges, achieved through gonadotropin receptor down-regulation and desensitization, has dramatically reduced the cycle cancellation rate to approximately 2% [2]. However, long-term GnRHa protocols are frequently associated with estrogen deprivation symptoms, such as hot flushes, sleep disturbances, and headaches, which commonly occur during the pre-stimulation phase [3].

Gonadotropin-releasing hormone antagonists (GnRH antagonists) competitively bind to the receptor, thereby inhibiting endogenous GnRH from stimulating pituitary cells. Ganirelix (also known as orgalutran), a synthetic third-generation GnRH antagonist, is a decapeptide derived from natural GnRH with amino acid substitutions at positions 1, 2, 3, 6, 8, and 10 [4]. By competitively blocking GnRH receptors in the pituitary gland, ganirelix inhibits pituitary hormone secretion, particularly LH, within hours of administration. Ganirelix acetate is typically administered via a subcutaneous injection of 0.25 mg daily, starting on day 5 or 6 of ovarian stimulation (when the dominant follicle reaches approximately 14 mm in diameter) and continuing until the day of triggering final follicular maturation with human chorionic gonadotropin (hCG). Pituitary LH and follicle-stimulating hormone (FSH) levels are fully restored within 48 h after discontinuing ganirelix.

The pharmacokinetics of ganirelix are favorable; a single subcutaneous injection of 0.25 mg in healthy female volunteers demonstrated a mean absolute bioavailability of 91.1% [5]. In clinical trials involving 463 patients receiving daily subcutaneous injections of 0.25 mg ganirelix, less than 1% experienced a premature LH surge before hCG administration [4]. In assisted reproductive technology (ART) settings, including IVF and intracytoplasmic sperm injection (ICSI), ganirelix acetate is extensively used in controlled ovarian stimulation (COS) protocols. Its use significantly shortens the duration of treatment, reduces the gonadotropin dosage required for stimulation, optimizes ovarian response, and enhances success rates. Additionally, ganirelix offers other clinical benefits, such as lowering the risk of severe ovarian hyperstimulation syndrome (OHSS) and mitigating estrogen deprivation symptoms commonly observed with GnRHa protocols [6, 7].

Despite the unique pharmacological benefits and promising applications of ganirelix acetate, its practical use is associated with potential safety concerns. Pooled data from three clinical trials involving 792 women undergoing IVF revealed that drug-related adverse reactions, including headache, malaise, and nausea, occurred in 2–3% of patients [8, 9]. Local reactions at the injection site (redness, bruising, pain, or itching) were reported in 12–20% of patients within one hour of injection, with 2% experiencing moderate or severe reactions. In a randomized, parallel clinical trial involving 45 healthy female volunteers of childbearing age, subcutaneous injections of 0.125, 0.25, or 0.5 mg ganirelix over a 7-day period resulted in headache (71%), injection site reactions (44%), and fatigue (24%) as the most commonly reported ADEs [10]. Similarly, a prospective, open-label, randomized clinical trial conducted in the United States to evaluate the safety of ganirelix acetate (0.25 mg) in women undergoing ART found that 28.4% (25/88) of patients reported post-treatment adverse events. The most frequently reported event was abdominal distension (12.5%), while four serious ADEs (OHSS, adnexal uterine pain, headache, and influenza) and one very serious event (ectopic pregnancy) were also documented [11]. While most studies indicate a favorable safety profile for ganirelix treatment, it is important to acknowledge the limitations of clinical trials in detecting rare ADEs. These trials often involve strict inclusion criteria and relatively small sample sizes, which may not fully represent real-world populations. Furthermore, the short duration of most clinical trials may not capture long-term safety outcomes, a critical consideration for drugs used in fertility treatments that require extended monitoring and evaluation.

Pharmacovigilance plays a critical role in the assessment, monitoring, and prevention of adverse drug events (ADEs) [12]. By collecting real-world data, spontaneous ADE reporting systems enable the identification of safety concerns that may not have been detected during clinical trials. Additionally, these systems provide foundational data for long-term safety evaluations of drugs and contribute to the global regulation of drug safety [13]. Two widely utilized spontaneous adverse event reporting databases—the U.S. Food and Drug Administration Adverse Event Reporting System (FAERS) and the Japanese Adverse Drug Event Report (JADER)—have recently collected a substantial number of adverse event reports from diverse populations (predominantly North America for FAERS and Japan for JADER) [13]. These databases serve as invaluable resources for the early detection and identification of potential adverse reactions. Researchers can leverage these data to facilitate continuous monitoring of ADEs through pharmacovigilance studies, which provide critical evidence for regulatory agencies to issue drug warnings, update product labeling, and enhance drug safety protocols [14]. Zou et al. recently utilized the FAERS and JADER databases to analyze and compare adverse drug events associated with GnRH agonists, identifying both known and novel ADEs not listed in the drug label [15]. Although ganirelix acetate was first approved by the Food and Drug Administration (FDA) in 1999, large-scale, real-world studies on its safety remain scarce. To address this gap, we conducted pharmacovigilance analyses using data from the FAERS and JADER databases. Employing a manifold analysis approach, we visualized the safety profile of ganirelix acetate across two distinct cohorts. Furthermore, we identified unexpected signals not listed in the current drug label and determined specific timeframes for the occurrence of ADEs. This pharmacovigilance study systematically analyzed the adverse drug events associated with ganirelix acetate across two independent cohorts. Our findings provide valuable insights to optimize the clinical use of ganirelix acetate, inform safe prescribing practices, and promote its rational application in assisted reproductive technologies.

## Materials and methods

### Data sources

In this retrospective, observational pharmacovigilance analysis, we utilized ADE data from the FAERS and JADER databases. The FAERS database (accessible at [https://fis.fda.gov/extensions/FPD-QDE-FAERS/FPD-QDE-FAERS.html]) provides quarterly data files that include seven datasets covering various aspects of ADE reporting: demographics (DEMO), drug characteristics (DRUG), indications (INDI), adverse events (REAC), patient outcomes (OUCT), source of report (RPSR), and duration of therapy (THER) [16]. In the FAERS architecture, these files were linked through specific identifiers, such as PRIMARYIDs [17]. The JADER database (accessible at [https://www.pmda.go.jp/index.html]) comprises four primary datasets: “Basic Information (DEMO)”, “Drug Information (DRUG)”, “Adverse Event Information (REAC)”, and “Primary Illness Information (HIST)“ [18]. Since ganirelix acetate was approved by the FDA in July 1999 and by the PMDA in January 2009. The study period for the data retrieval from FAERS was from January 2004 to June 2024 [using the generic name (GANIRELIX ACETATE) and brand names (ORGALUTRAN/ANTAGON)], and from January 2009 to March 2024 for JADER (using the search terms “ガニレリクス酢酸塩”).

### Data processing

Since the reports in both FAERS and JADER are spontaneously submitted, duplicates are common. To maintain data integrity and reliability, we meticulously follow the official data cleaning guidelines outlined by the U.S. FDA, ensuring the distinctiveness of the reports in compliance with high standards of scientific rigor. In FAERS, we identified and deleted duplicates reports by sorting the PRIMARYID (unique report identifier), CASEID (number for identifying a FAERS case), and FDA_DT (date FDA received the case) fields in the DEMO table. Specifically, for each CASEID, we retained the report with the most recent FDA_DT, and in cases where both CASEID and FDA_DT were identical, the report with the largest PRIMARYID was kept [19]. Each case report was assigned a unique PRIMARYID, with higher values corresponding to more recently submitted reports [20]. Since the first quarter of 2019, each quarterly data package has included a list of deleted reports [21]. After performing data deduplication, reports were excluded based on the CASEID listed in the deleted reports list. This rigorous methodology effectively eliminated redundant entries and enhanced the integrity of subsequent analyses. In JADER, we removed duplicates from the DRUG and REAC tables, and the DEMO table was linked to the DRUG and REAC tables using unique scenarios identified in the dataset [22]. Furthermore, since this study focuses solely on the safety of ganirelix in females, we selected the code for patient’s sex as “F” (female) in the FAERS database. In the JADER database, we utilized the gender identifier field to extract reports related to females. Records with missing, unknown, or inconsistent gender information were excluded from the analysis.

Drug role types were extracted from both FAERS and JADER. In FAERS, the roles included “primary suspect drug,” “secondary suspect drug,” “concomitant drug,” and “interaction.” In JADER, the roles were categorized as “primary suspect drug,” “concomitant drug,” and “interaction.” To improve the accuracy of the analysis, only ADE reports where the role_cod was “PS” (primary suspect) were included in the DRUG file.

All ADEs in FAERS were coded using the preferred terms (PT) from the standardized Medical Dictionary for Regulatory Activities (MedDRA) version 27.0, which organizes terms into five hierarchical levels: system organ class (SOC), high-level group term (HLGT), high-level term (HLT), preferred term (PT), and lowest-level term (LLT). MedDRA was also used to categorize ADEs in each report to their corresponding SOC level. Similarly, in JADER, ADEs were coded using the Dictionary of Medical Terms for Regulatory Activities/Japanese version 27.0 (www.pmrj.jp/jmo/php/indexj.php). To ensure the comparability of the analysis results, considering the differences in reporting formats and data structures between FAERS and JADER, we applied the following methods: (1) Standardized coding system: We adopted a consistent coding system for medical terminology (MedDRA 27.0) to reduce biases in data analysis caused by differing database formats and ensure uniformity in the terms used for adverse reactions across both databases. (2) Removal of duplicates and errors: We excluded duplicate and erroneous records from the spontaneous reporting data within the databases to minimize inconsistencies and enhance the completeness and reliability of the analysis. (3) Bayesian algorithms: We utilized Bayesian-based algorithms along with empirical Bayesian geometric averaging, which adapt to variations in data distributions and structures. This approach ensures consistent and reliable signals, thereby maintaining the comparability of results. (4) Timeframe selection: We chose the proper periods in both databases to minimize any biases linked to reporting during the clinical trial phase.

### Statistical analysis

Disproportionality analysis is a crucial method in pharmacovigilance used to identify potential associations between drugs and adverse events. In pharmacovigilance analysis, a signal refers to a statistically significant association between a given drug and a specific adverse event [23]. In this study, we applied four methods of disproportionality analysis: Reporting Odds Ratio (ROR), Proportional Reporting Ratio (PRR), Bayesian Confidence Propagation Neural Network (BCPNN), and Multi-Item Gamma Poisson Shrinker (MGPS) (Table 1). ROR is a widely used signal detection tool in pharmacovigilance that calculates the reporting odds ratio to assess the association between a drug and adverse events. It is effective in large-scale spontaneous reporting databases [24]. PRR compares the risk ratio of a specific drug with a control group, allowing better control of varying drug usage frequencies, though small denominators can cause result fluctuations [25]. BCPNN combines Bayesian theory and artificial neural networks, effectively managing complex probabilistic models and high-dimensional data, with the advantage of quantifying uncertainty for more stable results [26]. MGPS uses empirical Bayesian shrinkage estimation to reduce false positives and is particularly robust for rare events and small sample sizes, improving signal detection accuracy [27]. Frequency-based methods, such as ROR and PRR, are computationally simple and sensitive but prone to false positives when the number of ADEs is small [28]. Bayesian methods, such as BCPNN and MGPS, excel in integrating diverse data sources, reducing false positives, and detecting rare event signals, although they are computationally intensive [29]. While no gold standard has been established, this study combines four algorithms and conducts cross-validation, maximizing the advantages of each algorithm, validating results from various angles, and reducing the risk of false positives to enhance the detection of potential rare adverse events [30]. An ADE that meets the signal threshold of all four algorithms is considered a positive signal. In the FAERS, the time to onset (TTO) of ganirelix-associated ADEs was defined as the interval between the date of ADE onset (EVENT_DT) and medication initiation (START_DT). In the JADER database, both dates are recorded in the DRUG file. Reports missing medication initiation or ADE onset dates, or containing imprecise or inconsistent dates, were excluded. Descriptive statistics, including median, interquartile range, minimum, maximum, and Weibull shape parameters, were used to evaluate TTOs. Changes in ADE risk over time were assessed using the Weibull distribution test [31]. When the shape parameter (β) and its 95% confidence interval (CI) are below 1, the risk decreases over time, indicating an “early-failure” pattern. A β approximately equal to 1 with a CI including 1 suggests persistent risk (“random-failure”), while a β greater than 1 with a CI excluding 1 indicates increasing risk (“wear-out failure”) [32]. The Kaplan-Meier curves and log-rank tests further compared cumulative incidence across PTs.

**Table 1:** A two-by-two contingency table and detailed formulas for disproportionality analysis

	Target adverse drug event	Non-target adverse drug event	Sums
Ganirelix	a	b	a + b
Non-ganirelix	c	d	c + d
Total	a + c	b + d	a + b + c + d
**Methods**	**Formula**		**Threshold**
ROR	$\:ROR=\frac{a/c}{b/d}$		$\:\text{a}\ge\:3$
	$$\:SE\left(lnROR\right)=\sqrt{(\frac{1}{a}+\frac{1}{b}+\frac{1}{c}+\frac{1}{d})}$$		
	$\:95\text{\%}\text{C}\text{I}={\text{e}}^{\text{ln}\left(\text{R}\text{O}\text{R}\right)\pm\:1.96\text{s}\text{e}}$		$\:95\text{\%}\text{C}\text{I}\left(\text{l}\text{o}\text{w}\text{e}\text{r}\:\text{l}\text{i}\text{m}\text{i}\text{t}\right)>1$
PRR	$\:\text{P}\text{R}\text{R}=\frac{a/(a+b)}{c/(c+d)}$		$\:\text{a}\ge\:3$
	χ2=[(ad-bc)^2](a + b + c + d)/[(a + b)(c + d)(a + c)(b + d)]		$\:{\upchi\:}2\ge\:4$
BCPNN	$\:\text{I}\text{C}={\text{log}}_{2}\frac{p(\text{x},\text{y})}{p\left(\text{x}\right)p\left(\text{y}\right)}={\text{log}}_{2}\frac{\text{a}(\text{a}+\text{b}+\text{c}+\text{d})}{(\text{a}+\text{b})(\text{a}+\text{c})}$		$\:\text{I}\text{C}025>0$
	$$\:\text{E}\left(\text{I}\text{C}\right)={\text{log}}_{2}\frac{(\text{a}+{\upgamma\:}11)(\text{a}+\text{b}+\text{c}+\text{d}+{\upalpha\:})(\text{a}+\text{b}+\text{c}+\text{d}+{\upbeta\:})}{(\text{a}+\text{b}+\text{c}+\text{d}+{\upgamma\:})(\text{a}+\text{b}+{\upalpha\:}1)(\text{a}+\text{c}+{\upbeta\:}1)}$$		
	$${\rm{V}}\left( {{\rm{IC}}} \right) = {1 \over {{{\left( {\ln 2} \right)}^2}}}\left[ \matrix{{{\left( {{\rm{a}} + {\rm{b}} + {\rm{c}} + {\rm{d}}} \right) - {\rm{a}} + {\rm{\gamma }} - {\rm{\gamma }}11} \over {\left( {{\rm{a}} + {\rm{\gamma }}11} \right)\left( {1 + {\rm{a}} + {\rm{b}} + {\rm{c}} + {\rm{d}} + {\rm{\gamma }}} \right)}} + {{\left( {{\rm{a}} + {\rm{b}} + {\rm{c}} + {\rm{d}}} \right) - \left( {{\rm{a}} + {\rm{b}}} \right) + {\rm{a}} - {\rm{\alpha }}1} \over {\left( {{\rm{a}} + {\rm{b}} + {\rm{\alpha }}1} \right)\left( {1 + {\rm{a}} + {\rm{b}} + {\rm{c}} + {\rm{d}} + {\rm{\alpha }}} \right)}} \hfill \cr + {{\left( {{\rm{a}} + {\rm{b}} + {\rm{c}} + {\rm{d}} + {\rm{\alpha }}} \right) - \left( {{\rm{a}} + {\rm{c}}} \right) + {\rm{\beta }} - {\rm{\beta }}1} \over {\left( {{\rm{a}} + {\rm{b}} + {\rm{\beta }}1} \right)\left( {1 + {\rm{a}} + {\rm{b}} + {\rm{c}} + {\rm{d}} + {\rm{\beta }}} \right)}} \hfill \cr} \right]$$		
	$$\:{\upgamma\:}={\upgamma\:}11\frac{(\text{a}+\text{b}+\text{c}+\text{d}+{\upalpha\:})(\text{a}+\text{b}+\text{c}+\text{d}+{\upbeta\:})}{(\text{a}+\text{b}+{\upalpha\:}1)(\text{a}+\text{c}+{\upbeta\:}1)}$$		
	$$\:\text{I}\text{C}-2\text{S}\text{D}=\text{E}\left(\text{I}\text{C}\right)-2\sqrt{\text{V}\left(\text{I}\text{C}\right)}$$		
EBGM	$\:\text{E}\text{B}\text{G}\text{M}=\frac{a(a+b+c+d)}{(a+c)(a+b)}$		$\:\text{E}\text{B}\text{G}\text{M}05>2$
	$$\:\text{S}\text{E}\left(\text{l}\text{n}\text{E}\text{B}\text{G}\text{M}\right)=\sqrt{(\frac{1}{\text{a}}+\frac{1}{\text{b}}+\frac{1}{\text{c}}+\frac{1}{\text{d}})}$$		
	$$\:95\text{\%}\text{C}\text{I}={\text{e}}^{\text{ln}\left(\text{E}\text{B}\text{G}\text{M}\right)\pm\:1.96\text{s}\text{e}}$$		

Methods, formulas, and thresholds for calculating reporting odds ratio (ROR), Proportional Reporting Ratio (PRR), Bayesian Confidence Propagation Neural Network (BCPNN), and Empirical Bayesian Geometric Mean (EBGM). Variable ‘a’ denotes the number of individuals who experience target adverse events after exposure to target drug, variable ‘b’ represents the number of individuals who experience non-target adverse event following target drug exposure, variable ‘c’ indicates the number of individuals experiencing target adverse event after exposure to non-target drug, and variable ‘d’ refers to the number of individuals experiencing non-target adverse event following non-target drug exposure. 95% CI, 95% confidence interval; χ2, chi-squared; IC, information component; IC025: Information Component 2.5th percentile. E(IC), IC expectations; V(IC), variance of IC; EBGM, empirical Bayesian geometric mean; EBGM05, lower limit of 95% CI of EBGM

**Fig. 1:**
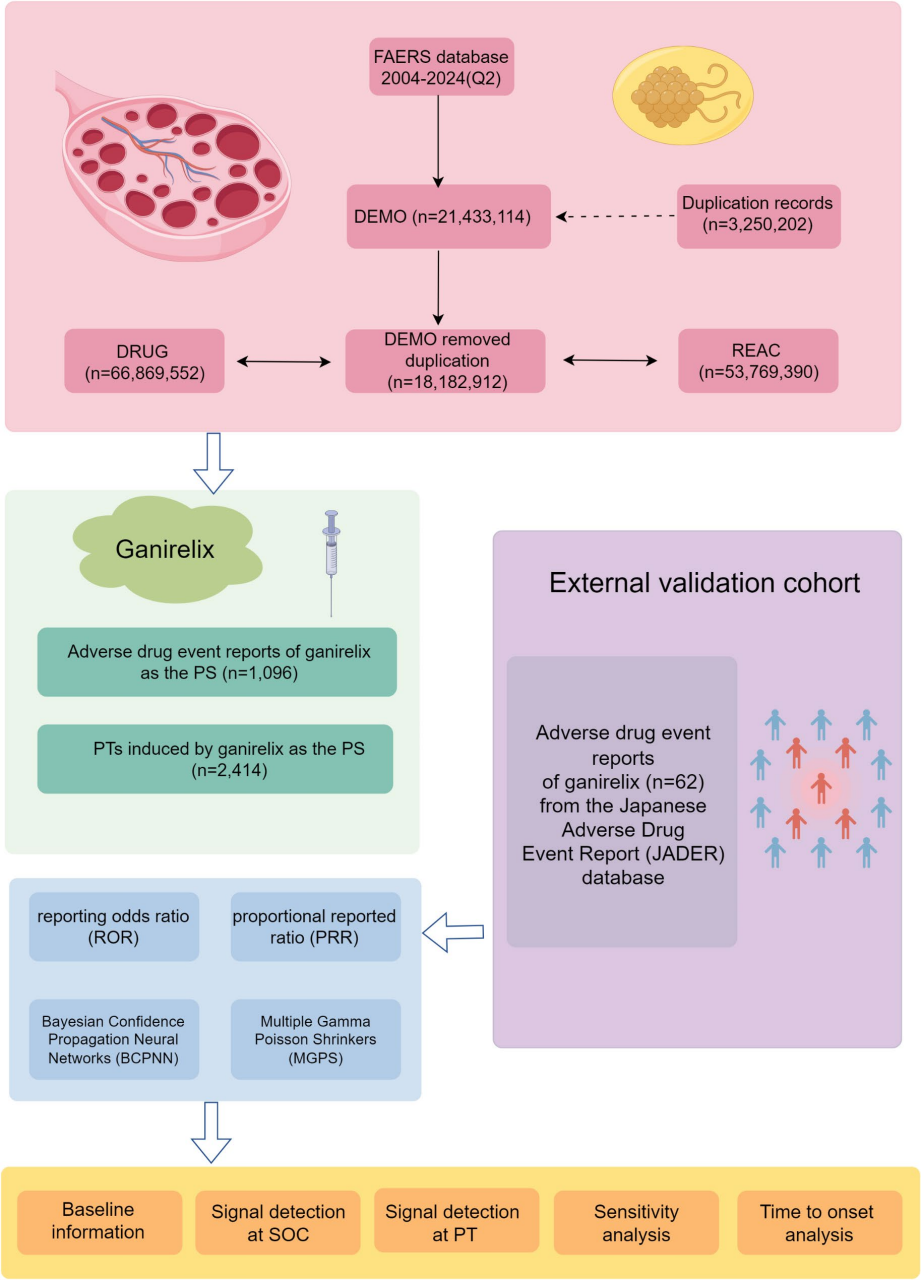
A flowchart illustrating the entire study, encompassing the following key aspects: data collection and cleaning processes for the two cohorts (FAERS and JADER), the methods of disproportionality analysis, and the critical components of the study. FAERS, FDA Adverse Event Reporting System; JADER, Japanese Adverse Drug Event Report; Q2, the second quarter; PT, preferred term; PS, primary suspect; SOC, system organ class; PT, preferred term

Data processing and analysis were performed using R (version 4.2.1) and Microsoft Excel 2019. Figure 1 outlines the study’s key steps and main components.

### Sensitivity analysis

Given that some adverse events may be associated with the underlying disease itself [33], and that the presence of these signals could potentially interfere with the true association between ganirelix and its adverse events, we performed a sensitivity analysis on the patient population receiving drug therapy. Considering the similarity in indications between cetrorelix and ganirelix [34], we further re-evaluated the signal strength for cetrorelix to serve as a comparative reference for ganirelix. With reference to previously published literature, all the positive signals identified in ganirelix were categorized as follows: signals consistent with the adverse events listed in the drug label were classified into the “expected with signal” group; signals that were positive in both ganirelix and cetrorelix were categorized as the “disease-expected” group; and the remaining signals that did not meet the criteria for the above two categories were classified as the “unexpected” group [35].

## Results

### Basic characteristics of ganirelix-related ADEs

**Fig. 2:**
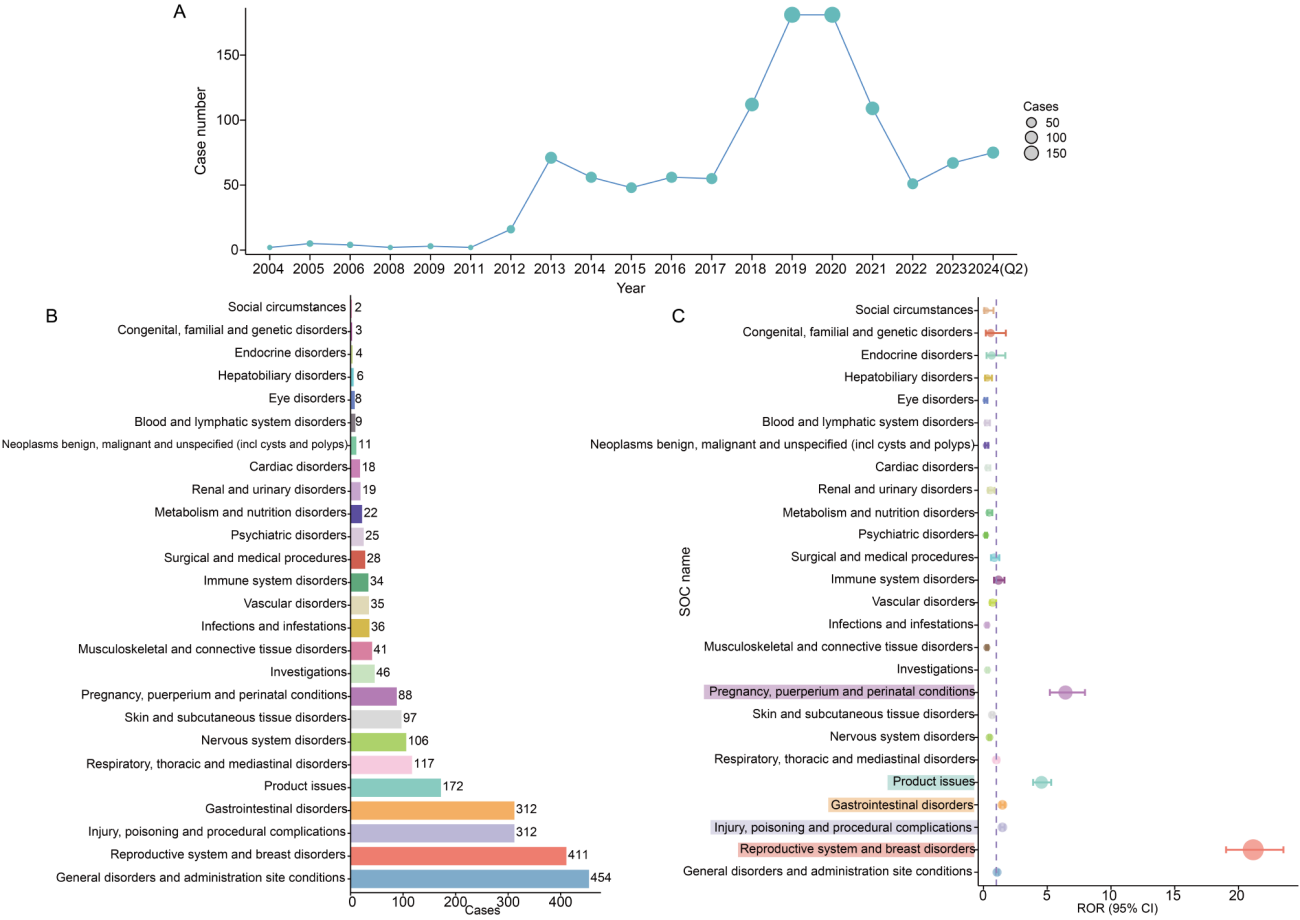
Signal detection at the SOC level. (**A**) A line graph illustrating the annual trend in the number of reports for ganirelix. (**B**) A distribution plot showing the number of reports categorized by SOC. (**C**) A forest plot displaying signal strength at the SOC level, represented by ROR values along with their corresponding 95% confidence intervals. Five SOCs that meet the positive signal threshold based on the ROR algorithm are highlighted for emphasis

From January 2004 to June 2024, a total of 1,096 ganirelix-related ADEs were identified in the FAERS database, with 2,414 PTs linked to ganirelix as the PS. Figure 2A illustrates the annual trend in ADE reports. Between 2004 and 2020, the number of reports showed a consistent increase, peaking in 2019 and 2020 (*n* = 181 for both years). From 2020 to 2024, the trend fluctuated, initially declining and then rising again. Table 2 provides baseline characteristics of these ADEs. Among reports with age information, individuals aged 18–65 years represented the largest group (*n* = 662, 60.4%). Detailed weight information was missing in the majority of reports (*n* = 937, 85.5%). The United States accounted for over half of the ADE reports (*n* = 597, 54.5%), followed by Germany (16.3%) and France (11.6%). Most reports were submitted by health professionals (*n* = 724, 66.1%), enhancing the reliability of the data. The most common serious outcome was hospitalization (initial or prolonged), accounting for 32.8% of reports (*n* = 359), while death was reported in only 2 cases (0.2%). Among the top five reported indications, “Female infertility” was the most frequently reported (*n* = 187, 17.1%), followed by in vitro fertilization, assisted reproductive technology, infertility, and prevention of premature ovulation (Table 2).

**Table 2:** Basic information on ADEs related to ganirelix from the FAERS database

Characteristics	Case number	Case proportion, %
**Sex**		
Female	1096	100%
**Age** < 18 years	1	0.1%
18–65 years	662	60.4%
Unknown	433	39.5%
**Weight** < 50 kg	26	2.4%
50–100 kg	129	11.8%
> 100 kg	51	0.5%
Unknown	937	85.5%
**Reported Countries (top five)** US	597	54.5%
DE	179	16.3%
FR	127	11.6%
JPN	50	4.6%
NL	35	3.2%
**Reported person** Health professionals	724	66.1%
Consumer	362	33.0%
Unknown	10	0.9%
**Outcome**		
HO	359	32.8%
LT	15	1.4%
DS	5	0.5%
DE	2	0.2%
OT	143	13.0%
CA	1	0.1%
Unknown	571	52.1%
**Indication (top five)**		
Infertility female	187	17.1%
In vitro fertilization	149	13.6%
Assisted reproductive technology	136	12.4%
Infertility	85	7.8%
Prevention of premature ovulation	56	5.1%

US, United States; DE, Germany; FR, France; JPN, Japan; NL, Netherlands; HO, Hospitalization-initial or prolonged; LT, Life-threatening; DS, Disability; CA, Congenital anomaly; DE, Death; OT, Other serious outcome; RI, Required intervention

### Safety signal detection

The analysis of ganirelix-related ADE reports identified 26 SOCs. The top three SOCs, based on the number of cases, were “general disorders and administration site conditions” (*n* = 454), “reproductive system and breast disorders” (*n* = 411), and “injury, poisoning and procedural complications” (*n* = 312) (Fig. 2B). Using disproportionality analysis, five SOCs surpassed the signal detection threshold for the ROR method, including “reproductive system and breast disorders” (ROR 21.16 [19.03–23.53]), “injury, poisoning and procedural complications” (ROR 1.48 [1.31–1.66]), “gastrointestinal disorders” (ROR 1.48 [1.32–1.67]), “product issues” (ROR 4.55 [3.89–5.31]), and “pregnancy, puerperium and perinatal conditions” (ROR 6.43 [5.20–7.96]) (Fig. 2C). Three SOCs met the positivity thresholds for all four signal detection algorithms, including “reproductive system and breast disorders” (ROR 21.16, PRR 17.75, EBGM05 16.22, IC025 2.48), “product issues” (ROR 4.55, PRR 4.3, EBGM05 3.77, IC025 0.44), and “pregnancy, puerperium and perinatal conditions” (ROR 6.43, PRR 6.23, EBGM05 5.22, IC025 0.97) (Table 3).

**Table 3:** Signal detection at the SOC level in FAERS

Syetem organ class	Cases	ROR(95%CI)	PRR(χ^2^)	EBGM(EBGM05)	IC(IC025)
General disorders and administration site conditions	454	1.06 (0.96–1.18)	1.05 (1.42)	1.05 (0.97)	0.07 (-1.59)
Reproductive system and breast disorders	411	21.16 (19.03–23.53)	17.75 (6549.08)	17.72 (16.22)	4.15 (2.48)
Injury, poisoning and procedural complications	312	1.48 (1.31–1.66)	1.41 (41.61)	1.41 (1.28)	0.5 (-1.17)
Gastrointestinal disorders	312	1.48 (1.32–1.67)	1.42 (42.82)	1.42 (1.29)	0.51 (-1.16)
Product issues	172	4.55 (3.89–5.31)	4.3 (442.09)	4.29 (3.77)	2.1 (0.44)
Respiratory, thoracic and mediastinal disorders	117	1 (0.83–1.21)	1 (0)	1 (0.86)	0 (-1.66)
Nervous system disorders	106	0.47 (0.39–0.57)	0.49 (60.13)	0.49 (0.42)	-1.02 (-2.68)
Skin and subcutaneous tissue disorders	97	0.67 (0.55–0.82)	0.68 (15.37)	0.68 (0.57)	-0.55 (-2.22)
Pregnancy, puerperium and perinatal conditions	88	6.43 (5.2–7.96)	6.23 (388.81)	6.23 (5.22)	2.64 (0.97)
Investigations	46	0.31 (0.23–0.41)	0.32 (70.17)	0.32 (0.25)	-1.64 (-3.31)
Musculoskeletal and connective tissue disorders	41	0.26 (0.19–0.36)	0.28 (82.23)	0.28 (0.21)	-1.85 (-3.52)
Infections and infestations	36	0.27 (0.19–0.37)	0.28 (71.95)	0.28 (0.21)	-1.85 (-3.52)
Vascular disorders	35	0.71 (0.51–0.99)	0.71 (4.24)	0.71 (0.54)	-0.49 (-2.16)
Immune system disorders	34	1.17 (0.83–1.64)	1.17 (0.82)	1.17 (0.88)	0.22 (-1.44)
Surgical and medical procedures	28	0.85 (0.59–1.24)	0.85 (0.71)	0.85 (0.62)	-0.23 (-1.9)
Psychiatric disorders	25	0.18 (0.12–0.27)	0.19 (91.14)	0.19 (0.14)	-2.39 (-4.06)
Metabolism and nutrition disorders	22	0.45 (0.3–0.69)	0.46 (14.4)	0.46 (0.32)	-1.13 (-2.79)
Renal and urinary disorders	19	0.54 (0.35–0.86)	0.55 (7.16)	0.55 (0.38)	-0.87 (-2.53)
Cardiac disorders	18	0.33 (0.21–0.53)	0.34 (24.26)	0.34 (0.23)	-1.58 (-3.24)
Neoplasms benign, malignant and unspecified (incl cysts and polyps)	11	0.21 (0.12–0.38)	0.21 (32.85)	0.21 (0.13)	-2.24 (-3.9)
Blood and lymphatic system disorders	9	0.26 (0.13–0.5)	0.26 (19.06)	0.26 (0.15)	-1.94 (-3.6)
Eye disorders	8	0.15 (0.07–0.3)	0.15 (38.47)	0.15 (0.09)	-2.71 (-4.38)
Hepatobiliary disorders	6	0.3 (0.13–0.66)	0.3 (9.9)	0.3 (0.15)	-1.74 (-3.41)
Endocrine disorders	4	0.64 (0.24–1.71)	0.64 (0.8)	0.64 (0.28)	-0.64 (-2.31)
Congenital, familial and genetic disorders	3	0.56 (0.18–1.75)	0.56 (1.02)	0.56 (0.22)	-0.83 (-2.5)

FAERS: FDA Adverse Event Reporting System; ROR: reporting odds ratio; CI: confidence interval; PRR: proportional reporting ratio; χ²: chi-squared; IC: information component; IC025: Information Component 2.5th percentile; EBGM: empirical Bayes geometric mean; EBGM05: lower limit of the 95% CI of EBGM; SOC: system organ class

**Fig. 3:**
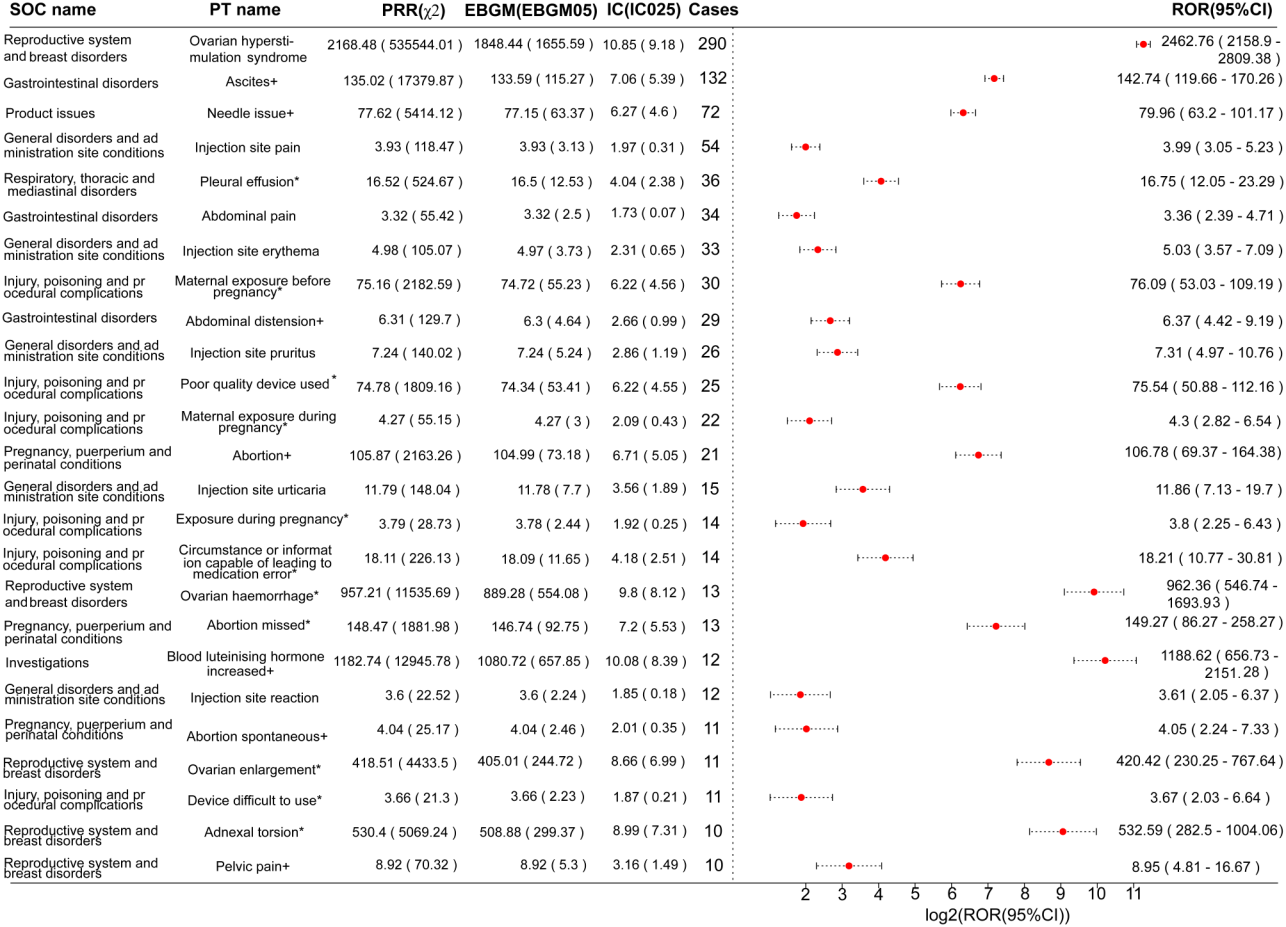
Signal detection at the PT level. The figure highlights all 25 positive PTs that meet the criteria of all four algorithms and have a report count of no less than 10. The left panel sequentially displays the SOC name, PT name, and signal strength generated by the different algorithms. On the right, the forest plot illustrates the log2-transformed ROR values along with their 95% confidence intervals. Asterisks indicate unexpected signals not listed in the drug label. SOC, system organ class; PT, preferred term

At the PT level, 65 PTs met the thresholds for all four algorithms (Table S1). PTs with higher reported cases included OHSS (*n* = 290, ROR 2462.76, PRR 2168.48, EBGM05 1655.59, IC025 9.18), ascites (*n* = 132, ROR 142.74, PRR 135.02, EBGM05 115.27, IC025 5.39), needle issue (*n* = 72, ROR 79.96, PRR 77.62, EBGM05 63.37, IC025 4.6), injection site pain (*n* = 54, ROR 3.99, PRR 3.93, EBGM05 3.13, IC025 0.31), and pleural effusion (*n* = 36, ROR 16.75, PRR 16.52, EBGM05 12.53, IC025 2.38). PTs with the strongest signal strength included OHSS, ovarian hemorrhage (*n* = 13, EBGM05 554.08), bladder tamponade (*n* = 4, EBGM05 311.57), adnexal torsion (*n* = 10, EBGM05 299.37), and uterine hyperstimulation (*n* = 4, EBGM05 298.91), suggesting a strong association with ganirelix use. Additionally, we identified several unexpected safety signals not listed in the drug label, including ovarian enlargement, ovarian abscess, hemoconcentration, ectopic pregnancy, maternal exposure before pregnancy, allergic dermatitis, and hyperkalemia, among others. Figure 3 highlights PTs with no fewer than 10 cases.

### Sensitivity analysis

When using cetrorelix as a comparator for disease-related adverse events, no reports were identified for certain adverse reactions, including pleural effusion, maternal exposure before pregnancy, ectopic pregnancy, oliguria, mood altered, allergic dermatitis, and bladder tamponade (Table S2). This indicates that these signals were specific to ganirelix. We identified several shared safety signals between ganirelix and cetrorelix, including ascites, needle-related issues, abdominal distension, abortion, increased blood luteinizing hormone, spontaneous abortion, post-procedural hemorrhage, pelvic pain, pelvic fluid collection, and device-associated injury. These adverse events are likely attributable to the underlying infertility condition rather than being solely caused by ganirelix itself.

To eliminate the influence of concomitant medications on the results, we excluded all adverse event reports associated with concomitant use of ganirelix and performed the disproportionality analysis. From 373 ADE reports involving ganirelix alone, persistent positive signals included needle issue, injection site pain, injection site erythema, ovulation disorder, allergic dermatitis, and abortion (Table S3).

### Time to onset analysis

**Fig. 4:**
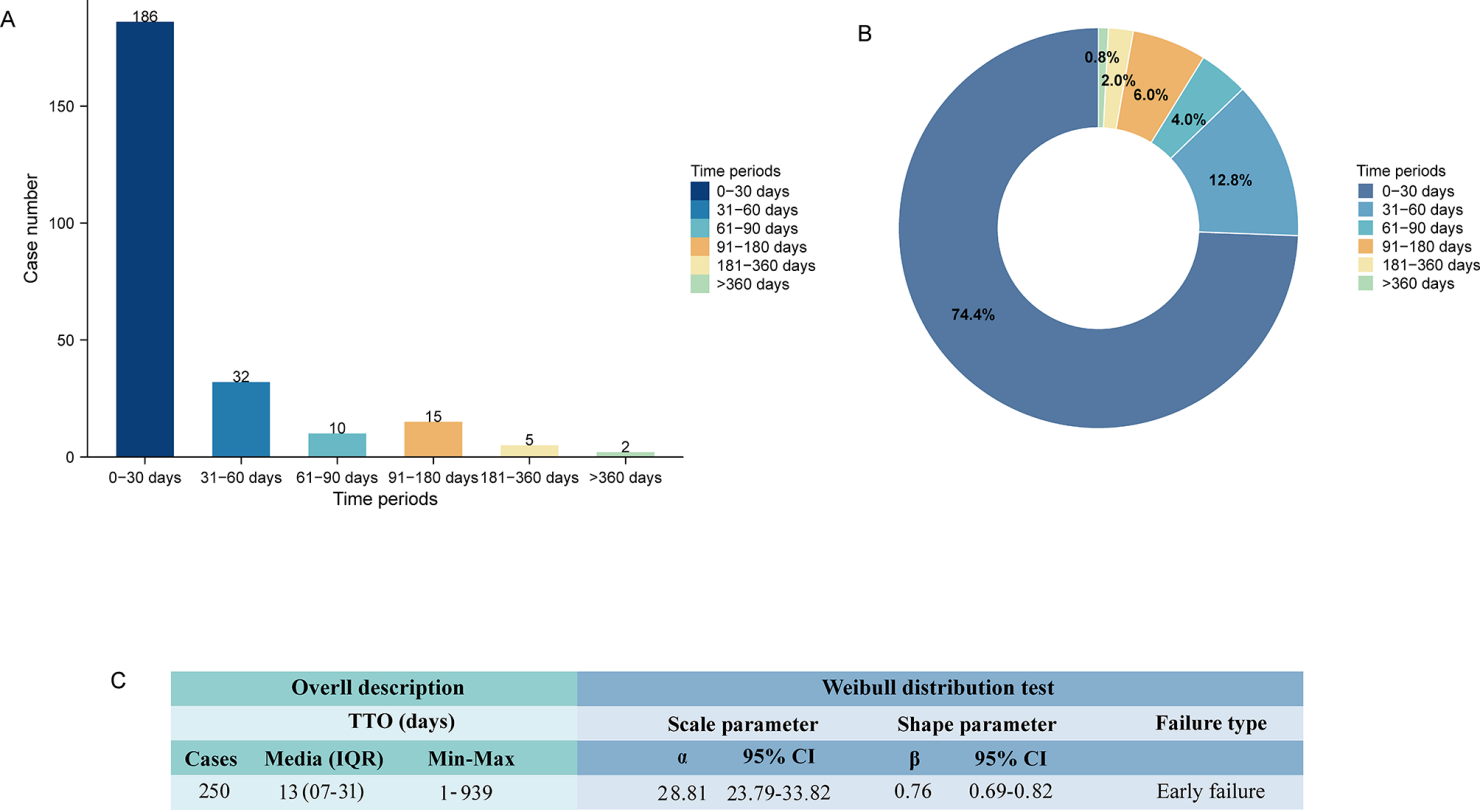
Time to onset (TTO) analysis (counted in days) of ganirelix-related ADEs at the overall level. (**A**) The frequency bar chart illustrates the distribution of TTO reports across various time periods. (**B**) The fan charts illustrate the percentage distribution of TTO reports across different time periods. (**C**) A comprehensive overview and Weibull distribution analysis of the 250 TTO reports are provided. Min, minimum; Max: maximum; IQR, interquartile range

We collected a total of 250 ADE reports with valid TTO data. The majority of these ADEs occurred within the first month following ganirelix administration (*n* = 186, 74.4%), followed by occurrences within 31–60 days (*n* = 32, 12.8%) (Fig. 4A and B). The median TTO for these events was 13 days (interquartile range [IQR]: 7–31) (Fig. 4C). The Weibull distribution analysis yielded a shape parameter (β) upper limit of 0.82, indicating an early-failure type (Fig. 4C). This suggests that, in general, the incidence of these ADEs progressively decreased over time.

**Fig. 5:**
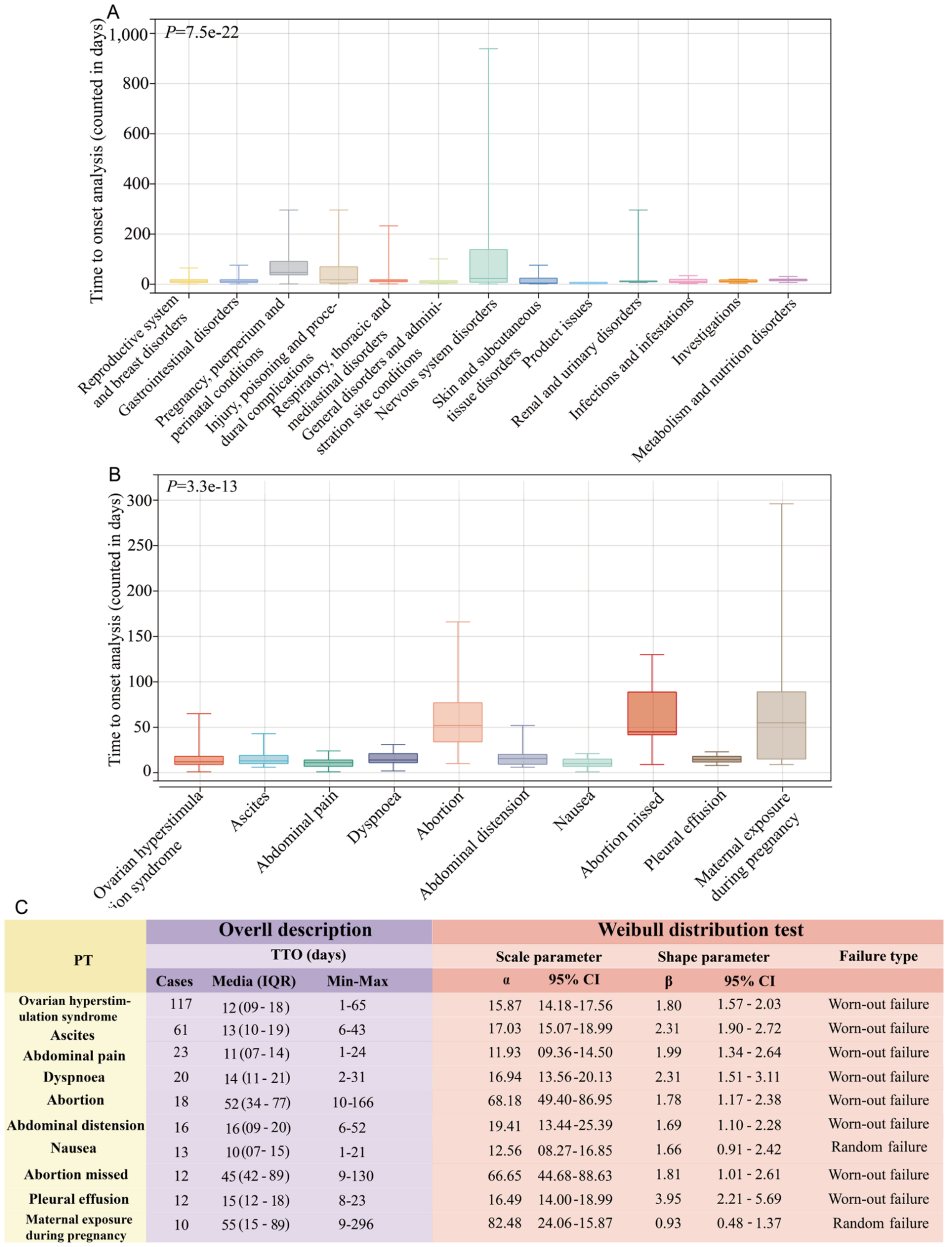
Time to onset (TTO) analysis at the SOC and PT level. (**A**) Box plot of the TTO at the SOC level for ganirelix. Bold bar within the stick: median TTO; Lower end of the stick: 1/4 quantile of the TTO; Upper end of the stick: 3/4 quantile of the TTO. (**B**) Box plot of the TTO at the PT level for ganirelix. We selected PTs with a minimum of 10 reports for detailed analysis. (**C**) A comprehensive overview and Weibull distribution analysis of the 10 PT TTO reports are presented. SOC, system organ class; PT, preferred term; Min, minimum; Max: maximum; IQR, interquartile range

At the SOC level, TTO analysis revealed that the median onset time (MOT) was less than 30 days for all SOCs except for “pregnancy, puerperium, and perinatal conditions,” which had a MOT of 47 days (Fig. 5A). Among these SOCs, three demonstrated MOTs of less than 10 days, including “general disorders and administration site conditions” (MOT: 9 days), “skin and subcutaneous tissue disorders” (MOT: 5 days), and “product issues” (MOT: 4.5 days). At the PT level, TTO analyses were conducted on PTs with no fewer than 10 reports. The PT with the shortest MOT was nausea (MOT: 10 days), while the PT with the longest MOT was maternal exposure during pregnancy (MOT: 55 days) (Fig. 5B). The Weibull distribution analysis further showed that two PTs, including nausea and maternal exposure during pregnancy, exhibited a random-failure type, indicating these events occurred consistently over time. In contrast, the remaining eight PTs demonstrated a wear-out failure type, reflecting an increasing probability of occurrence over time (Fig. 5C). Detailed results of the TTO analyses are presented in Table S4.

### External validation in JADER database

**Fig. 6:**
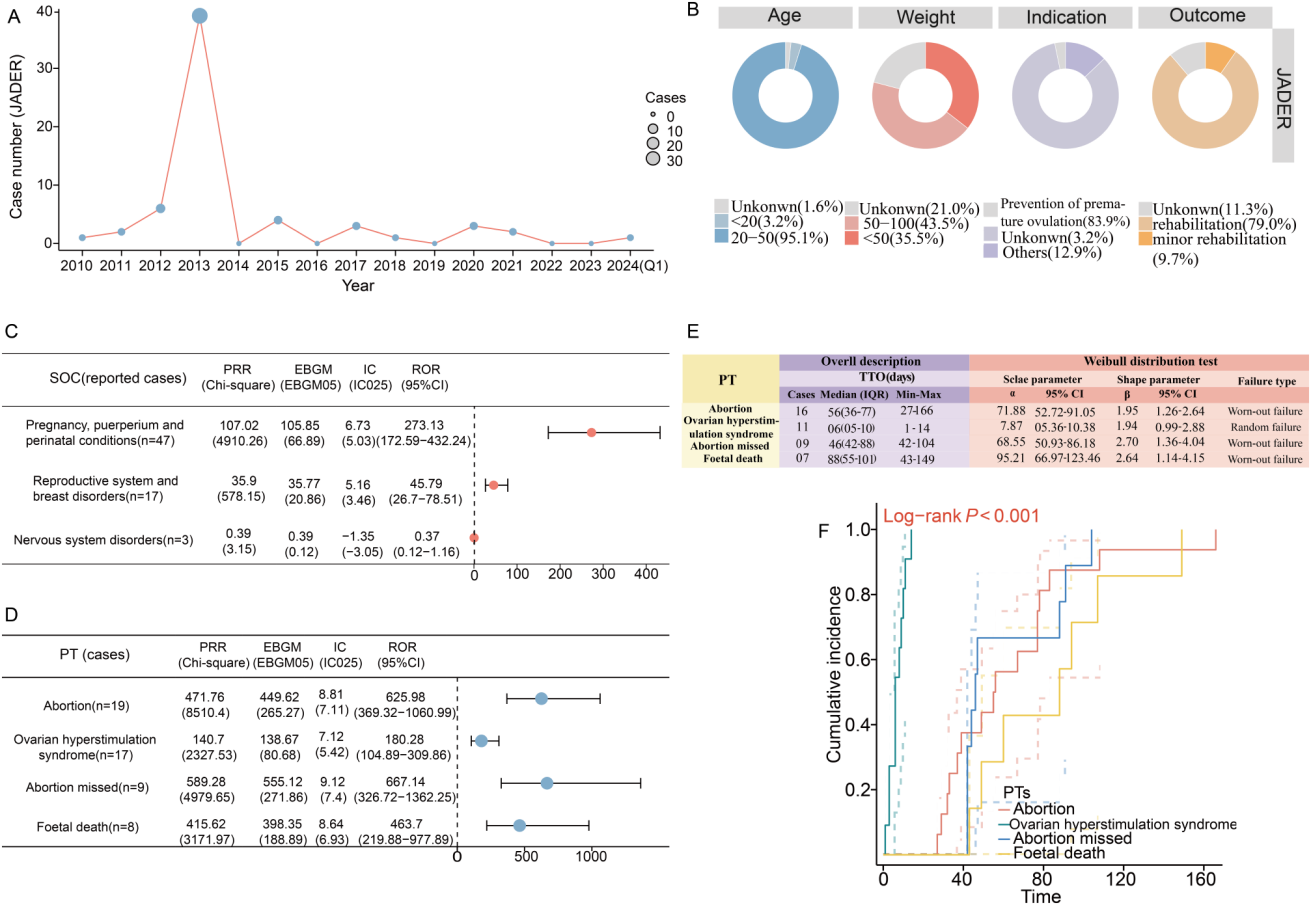
External validation from the JADER database. (**A**) Annual distribution of adverse drug event (ADE) reports spanning from 2010 to the first quarter of 2024. (**B**) Baseline information for ADE reports for ganirelix. (**C**) Signal detection at the SOC level: A forest plot representing reporting odds ratio (ROR) values along with their 95% confidence interval. (**D**) Signal detection at the PT level. The forest plot highlights the four positive PTs that meet the signal strength thresholds across all four methods. (**E**) Time to onset (TTO) analysis at the PT level. (**F**) The Kaplan-Meier curve depicts the cumulative incidence for the four PTs. JADER, Japanese Adverse Drug Event Report; SOC, system organ class; PT, preferred term; IQR, interquartile range

Between January 2009 to March 2024, a total of 62 reports of ganirelix-related ADEs were identified in JADER. The highest number of reports (*n* = 39) was recorded in 2013 (Fig. 6A). Baseline characteristics of the ADE reports are summarized in Fig. 6B, with the primary indication being the prevention of premature ovulation (83.9%). Signal detection at the SOC level identified two SOCs that met the positive thresholds of four signal detection algorithms. These were “pregnancy, puerperium, and perinatal conditions” (*n* = 47, ROR: 273.13; PRR: 107.02; EBGM05: 66.89; IC025: 5.03) and “reproductive system and breast disorders” (*n* = 17, ROR: 45.79; PRR: 35.9; EBGM05: 35.77; IC025: 3.46) (Fig. 6C).

At the PT level, signal detection revealed four PTs with no fewer than three reports. These included abortion (*n* = 19), OHSS (*n* = 17), missed abortion (*n* = 9), and fetal death (*n* = 8). All the four PTs met the thresholds across all four algorithms (Fig. 6D). TTO analysis at the PT level showed that OHSS had the shortest MOT of 6 days, with a random-failure type. In contrast, the other three PTs—abortion, missed abortion, and fetal death—had MOTs exceeding 45 days and exhibited a wear-out failure type (Fig. 6E). The Kaplan-Meier curve in Fig. 6F illustrated the cumulative incidence of ganirelix-related ADEs across these four PTs, revealing a significant difference in time-to-event distributions (log-rank *P* < 0.001).

## Discussion

### Signals consistent with drug label

Among the identified positive signals identified in our research, several were consistent with the drug label, including OHSS, injection site reactions, abdominal pain, and fetal death.

#### Ovarian hyperstimulation syndrome (OHSS)

Notably, OHSS demonstrated a high reporting frequency and strong signal strength in both the FAERS (*n* = 290; ROR 2462.76; PRR 2168.48; EBGM05 1655.59; IC025 9.18) and JADER (*n* = 17; ROR 180.28; PRR 140.70; EBGM05 80.68; IC025 5.42) databases, suggesting a close association between the occurrence of OHSS and the administration of ganirelix. OHSS is a serious complication of medically induced ovarian stimulation, characterized by ovarian enlargement, abdominal distension, ascites, pleural effusion, oliguria, hemoconcentration, and thromboembolic events [36]. The mild form of OHSS is reported to occur in 33% of IVF cycles, while the severe form is observed in 2–6% of cycles [37]. Despite the strong signal for OHSS in the pharmacovigilance data, it is important to recognize that a strong signal does not confirm a definitive causal relationship. In clinical practice, ganirelix is often used in a broader population that may include high-risk patients, such as those with polycystic ovary syndrome (PCOS) or those undergoing superovulation, who are inherently at a higher risk of developing OHSS [38].

Data from clinical trials provide mixed results on the incidence of OHSS with ganirelix. In a European Phase III clinical trial involving IVF patients, the incidence of OHSS was 2.4% in the ganirelix group compared to 5.9% in the buserelin group (a GnRH agonist) [39]. Similarly, a multicenter open-label study in Chinese women reported an OHSS incidence of 4.5% in the ganirelix group and 5.8% in the triptorelin group (another GnRH agonist) [40]. GnRH antagonists, including ganirelix, may reduce the risk of OHSS compared to GnRH agonists through mechanisms such as reduced local production of ovarian angiogenic factors and luteolysis [41, 42]. However, clinical trials conducted in North America, Europe, and the Middle East did not consistently confirm a reduction in OHSS incidence with ganirelix [43]. Moreover, a meta-analysis comparing GnRH antagonists and agonists reported that cetrorelix significantly reduced the risk of OHSS (OR = 0.23) compared to long-term GnRH agonist regimens, whereas ganirelix did not (OR = 1.13) [44]. Differences in patient populations, such as age, body mass index (BMI), and causes of infertility, may explain these inconsistent findings across clinical trials. Further studies are necessary to evaluate potential racial or ethnic variations in OHSS risk. Despite clinical evidence suggesting a potential reduction in OHSS risk with ganirelix, real-world pharmacovigilance data indicate that the risk remains, especially in high-risk populations. Factors such as the mode of administration, dosage, individual patient characteristics (e.g., PCOS, high ovarian responsiveness), and the concurrent use of other medications (e.g., gonadotropins, HCG) play significant roles in determining the risk of OHSS. Therefore, while ganirelix may lower the risk of OHSS compared to GnRH agonists, it does not entirely eliminate the risk in high-risk patients. Clinicians must remain vigilant, and further epidemiological studies are required to validate these findings and optimize the use of ganirelix in clinical practice.

#### Injection site reaction

We also identified a significant number of injection site reactions associated with ganirelix, including injection site pain (*n* = 54), injection site erythema (*n* = 33), injection site pruritus (*n* = 26), and injection site urticaria (*n* = 15), among others. While the FDA drug label for ganirelix mentions potential injection site adverse reactions, our analysis provides a more specific breakdown of these events. Historically, earlier generations of GnRH antagonists were known to cause mast cell degranulation upon contact, leading to localized histamine release [4]. However, studies on ganirelix have shown that it exhibits only minor histamine-releasing properties. Furthermore, subcutaneous administration of ganirelix has been reported to be relatively well tolerated [4]. Despite this, moderate or severe injection site reactions were observed in 12.4% of women receiving ganirelix in North American clinical trials within one hour of administration. Comparable rates were reported in European (11.9%) and Middle Eastern (24.1%) studies [43]. These findings suggest that injection site reactions to ganirelix are not uncommon. Evidence from pharmacokinetic studies also supports this, with injection site reactions consistently being the most frequently reported adverse effect. Notably, these reactions were generally mild in intensity and resolved spontaneously within 24 h after administration [4].

Data from a controlled, randomized, multicenter trial indicated that 16.6% of patients experienced at least one moderate or severe local reaction during ganirelix treatment. The most frequently reported reactions included moderate or severe skin redness (9.5%) and swelling (9.5%) observed one-hour post-injection [8]. Similarly, a randomized, double-blind clinical trial involving 333 women who received daily subcutaneous injections of ganirelix (0.0625–2 mg) for 4–5 days reported that 20.5% experienced at least one moderate injection site reaction, while 1.2% experienced severe local reactions. Skin erythema was the most commonly reported symptom, occurring in a dose-dependent manner, which underscores the potential link between ganirelix dosing and injection site adverse reactions [45]. Given these findings, clinicians should remain vigilant for potential injection site reactions following ganirelix administration to ensure patient safety, optimize treatment protocols, and enhance patient compliance. Early recognition and management of these adverse effects are critical to improving the overall treatment experience for patients.

#### Fetal death

The signal for fetal death was notably positive in both the FAERS (*n* = 6, ROR 21.05; PRR 21.00; EBGM05 10.72; IC025 2.72) and JADER (*n* = 8, ROR 463.70; PRR 415.62; EBGM05 398.35; IC025 6.93) databases. Additionally, clinical trials have reported a reduction in pregnancy rates during stimulation cycles using GnRH antagonists compared to long-term regimens with GnRH agonists [43]. Evidence suggests that GnRH antagonists may negatively affect oocyte quality, embryo development, or endometrial receptivity. Higher doses of GnRH antagonists have been associated with poorer embryo and oocyte quality, as well as reduced fertilization and implantation rates [45, 46]. Preclinical studies further support these concerns. For instance, in vitro studies demonstrated that high concentrations of GnRH antagonists completely blocked preimplantation embryonic development in mice [47]. Similarly, an animal experiment using GnRH antagonists in pregnant baboons found that two of three pregnancies resulted in stillbirths, while the third resulted in a live birth with low birth weight. During the treatment period, significant suppression of luteinizing hormone, estrone, and estradiol levels was observed, implicating GnRH antagonists in placental insufficiency and pregnancy loss early in gestation [48].

Based on these findings, we hypothesize that ganirelix may contribute to fetal death through the following mechanisms: (1) Reduction in sex hormone levels: Ganirelix inhibits gonadotropin secretion, leading to reduced ovarian production of estrogen and progesterone. Progesterone is crucial for maintaining endometrial integrity and supporting fetal development, and a decline in its levels may result in pregnancy failure [49]. (2) Endometrial changes: The recent discovery of GnRH receptors in human endometrial tissue suggests that GnRH antagonism or reduced sex hormone levels could induce endometrial atrophy or dysfunction. Such changes may impair embryo implantation and disrupt nutrient supply, ultimately leading to fetal death [50]. (3) Impairment of placental function: Estrogen and progesterone are essential for maintaining placental function. The reduction in these hormones caused by GnRH antagonists could lead to placental insufficiency, compromising the oxygen and nutrient supply to the fetus [48].

Our study also identified additional positive signals associated with ganirelix exposure before and during pregnancy. These include maternal exposure before pregnancy (*n* = 30; ROR 76.09; PRR 75.16; EBGM05 74.72; IC025 6.22), maternal exposure during pregnancy (*n* = 22; ROR 4.30; PRR 4.27; EBGM05 3.00; IC025 0.43), and exposure during pregnancy (*n* = 14; ROR 3.80; PRR 3.79; EBGM05 2.44; IC025 0.25). Such pregnancy-related exposures may pose potential risks to patient health. Given these findings, clinicians should take proactive measures to strengthen health education and improve patients’ understanding of proper drug use. Enhancing compliance with rational ganirelix use is essential to ensuring its safety and efficacy, particularly in high-risk patient populations. Further research and epidemiological studies are warranted to explore these risks in greater detail and to guide clinical practice.

### Disease-expected signals

To determine whether the identified positive signals are related to underlying infertility conditions, we compared patients using cetrorelix as a control group. Cetrorelix, approved in 1999, was one of the first clinically viable third-generation GnRH antagonists, and is clinically approved for use in women undergoing IVF with controlled ovarian stimulation to prevent premature LH surge [51]. Several common safety signals identified in both ganirelix and cetrorelix suggest that these adverse events may be associated with underlying infertility conditions, particularly abortion and spontaneous abortion, which are among the disease-expected signals. Similar positive signals (abortion and missed abortion) were also observed in the JADER database, further supporting this association.

Although the observed abortion signals may be related to the patients’ underlying infertility conditions, previous studies have suggested a potential association between the use of ganirelix and pregnancy loss. In a double-blind, randomized, dose-finding study evaluating the efficacy of ganirelix, the implantation rate was highest in the 0.25 mg group (21.9%) but lowest in the 2 mg group (1.5%). The rates of early miscarriage (within the first 6 weeks following embryo transfer) were reported to be 11.9% and 13% in the 1 mg and 2 mg groups, respectively [45]. These findings raise concerns regarding the impact of higher ganirelix doses on pregnancy outcomes. Previous research has shown that replacing GnRH agonists with GnRH antagonists can lead to unpredictable effects on estradiol production during follicular recruitment. For example, serum estradiol levels decreased prior to hCG administration in 35% of donor cycles and in 93% of donor cycles within 0.3 days of GnRH antagonist use. This unpredictable suppression of estradiol may impair the hormonal environment necessary for successful implantation and early pregnancy maintenance [52, 53]. Moreover, granulosa cells, endometrial cells, and embryos have been shown to express GnRH receptors [54]. Treatment with GnRH antagonist regimens has been linked to aberrant expression of molecular markers critical for endometrial receptivity. Specifically, GnRH antagonists may inhibit the expression of the c-kit receptor in endometrial stromal cells, disrupting stromal cell proliferation, which is essential for embryo implantation and early pregnancy support. This disruption may ultimately contribute to miscarriage and reduced clinical pregnancy rates [55, 56]. However, considering the limited number of ADE reports related to cetrorelix use in this study, the interpretation of these results should be approached with caution.

Although these observations suggest that GnRH antagonists may impact pregnancy outcomes through a certain mechanism, further studies are needed to clarify these effects in detail and establish more refined groupings to eliminate confounding factors related to patients’ underlying comorbidities. Future research should investigate the impact of GnRH antagonists on ovarian cells, oocytes, embryos, and the endometrium to better understand their potential role in pregnancy loss and optimize treatment protocols for assisted reproductive technologies.

### Unexpected signals not listed in the drug label

#### Ectopic pregnancy

Additionally, our study identified an unexpected and severe signal not listed in the drug label: ectopic pregnancy. In the FAERS database, ectopic pregnancy (*n* = 7; ROR 33.02; PRR 32.93; EBGM05 17.64; IC025 3.37) displayed strong signal strength, suggesting a significant correlation between ectopic pregnancy and ganirelix use. Similarly, in a clinical trial utilizing the GnRH antagonist cetrorelix for ovarian stimulation, ectopic pregnancies were reported in 3.4% (8 out of 231 participants) [57]. While ectopic pregnancy is a potentially life-threatening complication of ART, its incidence generally ranges between 2.1% and 8.6% [58, 59]. Emerging evidence indicates that GnRH and GnRH receptor (GnRHR) expression may play a role in ectopic pregnancies. Specifically, both GnRH and GnRHR have been identified in the trophoblast population and the tubal epithelium at the site of tubal ectopic pregnancies. Studies have demonstrated differences in GnRH expression between intrauterine endometrial and ectopic pregnancies, with GnRH signaling shown to stimulate extra-epithelial trophoblast invasion. This suggests that altered regulation of trophoblast function by GnRH may contribute to ectopic pregnancy [60]. Moreover, research by Zhang et al. demonstrated that patients receiving GnRH antagonist regimens exhibited increased expression of endometrial apoptosis-related molecules and decreased S100P protein levels in endometrial epithelial cells, potentially impairing endometrial receptivity [61]. These findings suggest that GnRH antagonists may disrupt the normal function of the endometrium, delay the window of implantation, and thereby increase the risk of ectopic pregnancy [62, 63]. Further evidence comes from a retrospective cohort study of 343 patients who underwent GnRH antagonist regimens and achieved clinical pregnancy [64]. This study identified GnRH agonist triggering as an independent risk factor for ectopic pregnancy in multifactorial analyses. Moreover, two separate reports highlighted a significant increase in ectopic pregnancy following GnRH agonist triggering [65]. Sahin et al. reported that GnRH agonist triggers resulted in a higher incidence of ectopic pregnancy compared to hCG triggers, while Sousa et al. observed ectopic pregnancies in 20% of patients following GnRH agonist triggers, significantly higher than the 1.6% incidence in patients who received hCG for ovulation induction [66].

These findings suggest that GnRH agonist triggering, often employed in GnRH antagonist regimens for individualized management (e.g., ovarian hyperresponsiveness), may transiently elevate estrogen levels, which in turn increase uterine contractions and tubal ciliary activity, thereby elevating the risk of ectopic pregnancy in susceptible patients [64]. However, it is essential to note that ectopic pregnancy is multifactorial, and other contributing factors include abnormal tubal function, prior pelvic inflammatory disease, smoking history, and the inherent risks associated with ART procedures. Given these risks, clinicians should exercise caution when using GnRH antagonists during ART. Close monitoring of pregnancies and timely ultrasound evaluations are critical to promptly detect and rule out ectopic pregnancies, ensuring patient safety. Further research is necessary to elucidate the underlying mechanisms and identify strategies to mitigate this risk in ART protocols.

#### Bladder tamponade

Despite the limited number of cases, bladder tamponade (*n* = 4) demonstrated a remarkably strong signal in the FAERS database (ROR 771.47, PRR 770.3, EBGM05 311.57, IC025 7.8). Currently, there are no direct studies investigating the causal relationship between GnRH antagonists and bladder tamponade. However, several findings suggest potential mechanisms by which GnRH antagonists, such as ganirelix, might influence bladder function. Interestingly, GnRH receptors have been detected in human prostate and bladder tissues, and messenger RNA for GnRH receptors has been identified in the urethra and forced urethral muscles of dogs [67, 68]. A potential link between GnRH signaling and urinary incontinence has also been proposed in this species [68]. While the exact role of GnRH receptor-mediated signaling in bladder function remains unclear, it is plausible that it could influence mechanical afferent pathways, neural signaling, or smooth muscle regulation. Supporting this hypothesis, a study by Russo et al. found that systemic administration of ganirelix counteracted experimental bladder detrusor overactivity in female rats [69]. Inhibition of detrusor overactivity allowed the bladder to return to a more normal state, increasing its ability to hold urine and expanding its filling volume. This observation aligns with the clinical manifestations of bladder tamponade, suggesting that GnRH antagonists could indirectly contribute to bladder dysfunction.

In addition, GnRH antagonists may affect bladder function through other mechanisms. First, they reduce estrogen levels, which are crucial for maintaining lower urinary tract function. A decrease in estrogen may lead to bladder smooth muscle dysfunction, impairing the bladder’s ability to effectively void urine [70]. Second, prolonged suppression of hormone levels can result in atrophy of the genitourinary system, which may further compromise storage and voiding functions. Bladder tamponade is a serious condition characterized by increased intravesical pressure, which can impair renal function if not promptly addressed. Given these findings, clinicians should be vigilant about the potential risk of bladder tamponade associated with ganirelix use. Monitoring bladder function during treatment and promptly addressing any urinary symptoms is essential to mitigate this risk. Further research is warranted to explore the exact mechanisms of bladder dysfunction and the potential relationship between GnRH antagonists and bladder tamponade.

#### Dermatitis allergic

GnRH antagonists are highly lipophilic compounds. Upon subcutaneous administration, they bind strongly to GnRH receptors on mast cells, potentially leading to catabolism and histamine release [43]. While first-generation GnRH antagonists were hindered in clinical development due to allergic side effects caused by histamine release, modern GnRH antagonists, such as ganirelix, have largely overcome these challenges [6]. Early safety studies reported no allergic reactions or antibody formation during the first five years of ganirelix’s use on the market [71]. However, in our study, we identified a signal for dermatitis allergic associated with ganirelix use (*n* = 4; ROR 7.98; PRR 7.97; EBGM05 3.5; IC025 1.33). Although most reports of injection site redness and itching were deemed non-allergic local reactions [71, 72], it is possible that the molecular structure of ganirelix or its excipients could be recognized by the immune system as foreign substances, triggering an immune response and resulting in allergic symptoms. Additionally, patient-specific factors, such as genetic predisposition or immune status, may influence sensitivity to ganirelix and increase the risk of allergic reactions. While anaphylactic reactions to ganirelix acetate remain rare, clinical vigilance is essential. It is critical to assess the risk of allergy in patients before treatment and to monitor for any signs of hypersensitivity during administration to ensure patient safety.

Several additional signals were observed in our analysis. Abdominal distension, pleural effusion, and ovarian enlargement were identified, but these are likely clinical manifestations of OHSS, a known complication of ART. However, we also observed other unexpected signals requiring further investigation: pigmentation disorder, cardiovascular disorder, mood alteration, premature baby, hyperkalemia. The relationship between these signals and ganirelix use remains unclear and warrants further exploration in future clinical trials. These findings highlight the need for ongoing pharmacovigilance and targeted studies to fully elucidate the safety profile of ganirelix and its potential off-target effects.

### Time to onset analysis

The identification of the timing of ADEs plays a crucial role in guiding health professionals and patients in risk prevention [73]. In drug safety analyses, the use of the Weibull distribution provides valuable insights into the patterns of ADE occurrence, helping inform clinical decision-making and risk management strategies [74]. Our TTO analyses revealed that, at the aggregate level, the median onset time of ganirelix-associated ADEs was 13 days, with an overall early-failure pattern. This indicates that most ADEs are more likely to occur shortly after starting ganirelix treatment. However, at the PT level, the majority of PTs displayed a worn-out failure pattern, characterized by an increasing likelihood of ADEs over time. This pattern is typically associated with prolonged drug use or physiological changes resulting from drug accumulation.

It is worth noting that ganirelix is usually administered during days 5–7 of follicular stimulation and continued until ovulation is triggered, typically over 3–7 days. With a half-life of approximately 10–20 h, ganirelix’s short treatment duration suggests that its long-term safety should still be closely monitored in clinical practice, particularly in patients with extended treatment courses or higher cumulative doses. Interestingly, certain ADEs showed different patterns. For example, nausea and maternal exposure during pregnancy exhibited a random failure pattern in the FAERS database, indicating that these events were not significantly related to the duration of ganirelix use or cumulative dose. Clinicians should remain vigilant about these adverse reactions, as they can occur at any point during treatment. Additionally, OHSS demonstrated differing patterns across databases: a worn-out failure type in FAERS and a random-failure type in JADER. These discrepancies may be attributed to the low number of reports in JADER, differences in database reporting practices, or even racial and demographic variations in response to the drug.

In conclusion, identifying the timing of ADEs through spontaneous reporting databases offers significant value for improving drug safety monitoring. It provides critical insights for guiding clinical use, managing risks, and informing future drug development. By understanding the patterns and timing of ADEs, clinicians can optimize treatment plans, anticipate potential complications, and ensure better patient outcomes.

### Limitations

Our study has several limitations that should be considered. First, FAERS and JADER are spontaneous reporting systems, which means that not all drug-related adverse events are reported, leading to potential issues such as underreporting, overreporting, duplicate reporting, and reporting bias. These limitations inherently affect the reliability and comprehensiveness of the data [28]. Second, the reports in these databases often lack essential information, including detailed medical histories, specific medication regimens, comorbidities, and co-administration of other drugs. These missing data represent confounding variables that are challenging to control and may compromise the accuracy and validity of the final outcome analysis [16]. Furthermore, despite conducting a sensitivity analysis, we cannot entirely rule out the possibility that the adverse events may be attributed to the indication for the drug rather than the drug itself. Third, our analysis relied on disaggregated data to estimate signal strength, which cannot establish causality between ganirelix use and ADEs. Furthermore, the absence of data on the total number of individuals exposed to ganirelix prevents the calculation of the incidence or frequency of ADEs, limiting the ability to quantify risks [75]. Fourth, the total number of cases reported in FAERS and JADER varied significantly, and the focus on data from two specific regions—the United States and Japan—may limit the generalizability of our findings. Differences in demographic characteristics, medical practices, and prescribing patterns across other populations were not accounted for and could influence the results [76].

To address these limitations, future research should employ more rigorous methodologies, such as prospective epidemiological studies or controlled clinical trials. Combining data from diverse sources and including larger, more heterogeneous populations would provide a more comprehensive and accurate assessment of ganirelix’s safety profile. Such efforts are essential to generate robust evidence, inform clinical decision-making, and improve patient outcomes.

## Conclusion

In this study, retrospective pharmacovigilance analyses using the FAERS and JADER real-world databases not only confirmed the known adverse reactions listed in the drug label for ganirelix but also identified several potential ADEs that have not yet been documented. Through TTO analysis, we were able to determine the precise timing of these adverse reactions, providing valuable insights into their occurrence patterns. The findings of this study serve as an important reference for ensuring the safe clinical use of ganirelix. However, given the inherent limitations of spontaneous reporting databases, including issues such as indication confounding, underreporting, and the inability to establish causality, these findings should be interpreted cautiously as hypothesis-generating results. Further prospective studies and long-term clinical trials are necessary to validate the results of this study.

## Electronic supplementary material

Below is the link to the electronic supplementary material.

Supplementary Material Table S1: The 65 PT entries that simultaneously satisfied the four methods of disproportionality analysis with positive signal strength in FAERS. The PT entries are categorized into three groups: expected with signal (unmarked), disease-expected (marked with **+**), and unexpected (marked with *), and are listed in descending order of case number.

Supplementary Material Table S2: Signal strength for adverse drug events with no less than three counts for cetrorelix as comparators for ganirelix.

Supplementary Material Table S3: Complete results of sensitivity analysis.

Supplementary Material Table S4: Results of a more detailed TTO analysis performed at both SOC and PT level. Min: minimum; Max: maximum; IQR: interquartile range; Q1: first quartile; Q3: third quartile; SD: standard deviation; SE: standard error.

## Data Availability

No datasets were generated or analysed during the current study.
